# 5-Iodo-2,7-dimethyl-3-phenyl­sulfonyl-1-benzofuran

**DOI:** 10.1107/S1600536808011240

**Published:** 2008-04-30

**Authors:** Hong Dae Choi, Pil Ja Seo, Byeng Wha Son, Uk Lee

**Affiliations:** a Department of Chemistry, Dongeui University, San 24 Kaya-dong Busanjin-gu, Busan 614-714, Republic of Korea; b Department of Chemistry, Pukyong National University, 599-1 Daeyeon 3-dong Nam-gu, Busan 608-737, Republic of Korea

## Abstract

The title compound, C_16_H_13_IO_3_S, was prepared by the oxidation of 5-iodo-2,7-dimethyl-3-phenyl­sulfanyl-1-benzofuran with 3-chloro­peroxy­benzoic acid. The phenyl ring makes a dihedral angle of 76.31 (8)° with the plane of the benzofuran fragment. The crystal structure is stabilized by aromatic π–π inter­actions between the furan and benzene rings of neighbouring mol­ecules, and between the benzene rings of neighbouring mol­ecules; the centroid–centroid distances within the stack are 3.700 (4) and 3.788 (4) Å. In addition, the crystal structure exhibits inter- and intra­molecular C—H⋯O inter­actions, and an I⋯O halogen bond with an I⋯O distance of 3.282 (2) Å and a nearly linear C—I⋯O angle of 165.69 (8)°.

## Related literature

For the crystal structures of similar 3-phenyl­sulfonyl-1-benzofuran compounds, see: Choi *et al.* (2008*a* ▶,*b* ▶). For a review of halogen bonding, see: Politzer *et al.* (2007 ▶).
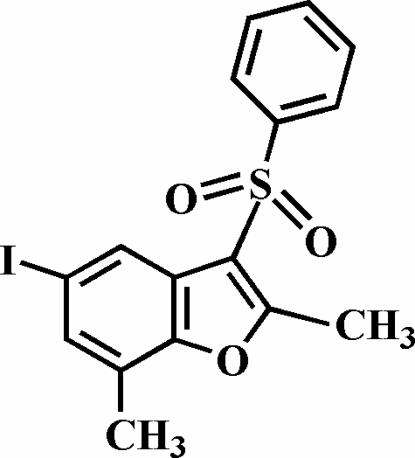


## Experimental

### 

#### Crystal data

C_16_H_13_IO_3_S
*M* *_r_* = 412.22
Monoclinic, 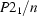

*a* = 8.1165 (5) Å
*b* = 14.0295 (9) Å
*c* = 13.2470 (8) Å
β = 90.320 (1)°
*V* = 1508.42 (16) Å^3^
*Z* = 4
Mo *K*α radiation
μ = 2.27 mm^−1^
*T* = 173 (2) K
0.40 × 0.20 × 0.20 mm

#### Data collection

Bruker SMART CCD diffractometer
Absorption correction: multi-scan (*SADABS*; Sheldrick, 2000 ▶) *T*_min_ = 0.579, *T*_max_ = 0.641
9110 measured reflections
3285 independent reflections
3069 reflections with *I* > 2σ(*I*)
*R*_int_ = 0.032

#### Refinement

*R*[*F* ^2^ > 2σ(*F* ^2^)] = 0.026
*wR*(*F* ^2^) = 0.074
*S* = 0.99
3285 reflections
193 parameters
H-atom parameters constrained
Δρ_max_ = 1.13 e Å^−3^
Δρ_min_ = −1.01 e Å^−3^

### 

Data collection: *SMART* (Bruker, 2001 ▶); cell refinement: *SAINT* (Bruker, 2001 ▶); data reduction: *SAINT*; program(s) used to solve structure: *SHELXS97* (Sheldrick, 2008 ▶); program(s) used to refine structure: *SHELXL97* (Sheldrick, 2008 ▶); molecular graphics: *ORTEP-3* (Farrugia, 1997 ▶) and *DIAMOND* (Brandenburg, 1998 ▶); software used to prepare material for publication: *SHELXL97*.

## Supplementary Material

Crystal structure: contains datablocks global, I. DOI: 10.1107/S1600536808011240/zl2112sup1.cif

Structure factors: contains datablocks I. DOI: 10.1107/S1600536808011240/zl2112Isup2.hkl

Additional supplementary materials:  crystallographic information; 3D view; checkCIF report

## Figures and Tables

**Table 1:** Hydrogen-bond geometry (Å, °)

*D*—H⋯*A*	*D*—H	H⋯*A*	*D*⋯*A*	*D*—H⋯*A*
C11—H11⋯O1^i^	0.95	2.59	3.382 (4)	141
C13—H13⋯O3^ii^	0.95	2.44	3.342 (4)	159
C14—H14⋯O2	0.95	2.58	2.931 (4)	103
C16—H16*B*⋯O3	0.98	2.48	3.191 (4)	129

Symmetry codes: (i) 
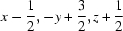
; (ii) 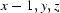.

**Fig. 1.**
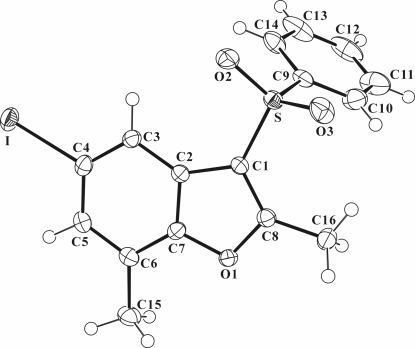
The molecular structure of the title compound, showing displacement ellipsoids drawn at the 50% probability level.

**Fig. 2.**
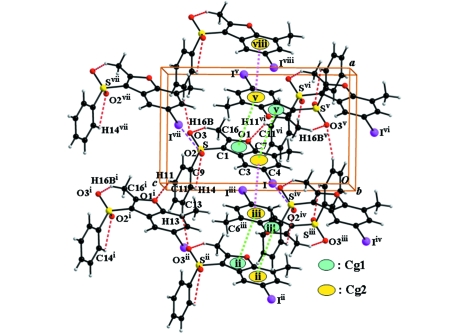
π—π interactions, C—H···O hydrogen bonds, and I···O halogen bond (dotted lines) in the title compound. Cg denotes the ring centroids. [Symmetry code: (i) x - 1/2, -y + 3/2, z + 1/2; (ii) x - 1, y, z; (iii) -x, -y + 1, -z + 1; (iv) x - 1/2, -y + 1/2, z - 1/2; (v) -x + 1, -y + 1, -z + 1; (vi) x + 1/2, -y + 3/2, z - 1/2; (vii) x + 1/2, -y + 1/2, z + 1/2; (viii) x + 1, y, z.]

## supplementary crystallographic information

### Comment

As a part of our ongoing studies on the synthesis and structure of 3-phenyl-sulfonyl-1-benzofuran analogues, the crystal structure of 5-bromo-2-methyl-3-phenylsulfonyl-1-benzofuran (Choi *et al.*, 2008*a*) and 2,5,7-trimethyl-3-phenylsulfonyl-1-benzofuran (Choi *et al.*, 2008*b*) have been recently described in the literature. Herein we report the molecular and crystal structure of the title ompound, 5-iodo-2,7-dimethyl-3-phenylsulfonyl-1-benzofuran (Fig. 1).

The benzofuran unit is essentially planar, with a mean deviation of 0.009 Å from the least-squares plane defined by the nine constituent atoms. The phenyl ring (C9—C14) makes a dihedral angle of 76.31 (8)° with the plane of the benzofuran fragment. The crystal packing (Fig. 2) is stabilized by two different π—π interactions within each stack of molecules; one between the furan ring (*Cg*1) and an adjacent benzene ring (*Cg*2^v^) of a benzofuran unit {distance 3.700 (4) Å}, and a second between the benzene ring (*Cg*2) and an adjacent benzene ring (*Cg*2^iii^) of the benzofuran unit {distance 3.788 (4) Å}(*Cg*1 and *Cg*2 are the centroids of the O1/C8/C1/C2/C7 furan ring and the C2—C7 benzene ring, respectively, symmetry code as in Fig. 2). The molecular packing is further stabilized by inter- and intramolecular C—H···O interactions (Table 1), and by a halogen bond (Politzer *et al.*, 2007) between the iodine atom and the oxygen atom of the S═O unit (Fig. 2; symmetry code as in Fig, 2).

### Experimental

3-Chloroperoxybenzoic acid (471 mg, 2.1 mmol) was added in small portions to a stirred solution of 5-iodo-2,7-dimethyl 3-phenylsulfanyl-1-benzofuran (380 mg, 1.0 mmol) in dichloromethane (30 ml) at 273 K. After being stirred for 4 h at room temperature, the mixture was washed with saturated sodium bicarbonate solution and the organic layer was separated, dried over magnesium sulfate, filtered and concentrated in vacuum. The residue was purified by column chromatography (hexane-ethyl acetate, 2: 1 *v*/*v*) to afford the title compound as a colorless solid [yield 78%, m.p. 431–432 K; *R*_f_ = 0.61 (hexane-ethyl acetate, 2:1 *v*/*v*)]. Single crystals suitable for X-ray diffraction were prepared by evaporation of a solution of the title compound in benzene at room temperature. Spectroscopic analysis: ^1^H NMR (CDCl_3_, 400 MHz) δ 2.41 (s, 3H), 2.80 (s, 3H), 7.42 (s, 1H), 7.50–7.32 (m, 3H), 7.98 (d, J = 7.32 Hz, 2H), 8.06 (s, 1H); EI—MS 412 [*M*^+^].

### Refinement

All H atoms were geometrically positioned and refined using a riding model, with C—H = 0.95 Å for aromatic H atoms, 0.98 Å for methyl H atoms, respectively, and with *U*_iso_(H) = 1.2Ueq(C) for aromatic H atoms and 1.5Ueq(C) for methyl H atoms. The highest peak in the difference map is 0.75 Å from I and the largest hole is 0.69 Å from I.

### Crystal data

**Table:**

C_16_H_13_IO_3_S	*F*_000_ = 808
*M**_r_* = 412.22	*D*_x_ = 1.815 Mg m^−^^3^
Monoclinic, *P*2_1_/*n*	Melting point = 431–432 K
Hall symbol: -P_2yn	Mo *K*α radiation λ = 0.71073 Å
*a* = 8.1165 (5) Å	Cell parameters from 6912 reflections
*b* = 14.0295 (9) Å	θ = 2.9–28.3º
*c* = 13.2470 (8) Å	µ = 2.27 mm^−^^1^
β = 90.320 (1)º	*T* = 173 (2) K
*V* = 1508.42 (16) Å^3^	Block, colorless
*Z* = 4	0.40 × 0.20 × 0.20 mm

### Data collection

**Table:**

Bruker SMART CCD diffractometer	3285 independent reflections
Radiation source: fine-focus sealed tube	3069 reflections with *I* > 2σ(*I*)
Monochromator: graphite	*R*_int_ = 0.032
Detector resolution: 10.0 pixels mm^-1^	θ_max_ = 27.0º
*T* = 173(2) K	θ_min_ = 2.9º
φ and ω scans	*h* = −10→7
Absorption correction: multi-scan(SADABS; Sheldrick, 2000)	*k* = −17→16
*T*_min_ = 0.579, *T*_max_ = 0.641	*l* = −16→16
9110 measured reflections	

### Refinement

**Table:**

Refinement on *F*^2^	Hydrogen site location: inferred from neighbouring sites
Least-squares matrix: full	H-atom parameters constrained
*R*[*F*^2^ > 2σ(*F*^2^)] = 0.027	*w* = 1/[σ^2^(*F*_o_^2^) + (0.0428*P*)^2^ + 1.8205*P*] where *P* = (*F*_o_^2^ + 2*F*_c_^2^)/3
*wR*(*F*^2^) = 0.074	(Δ/σ)_max_ = 0.001
*S* = 0.99	Δρ_max_ = 1.13 e Å^−^^3^
3285 reflections	Δρ_min_ = −1.01 e Å^−^^3^
193 parameters	Extinction correction: SHELXL97 (Sheldrick, 2008), Fc^*^=kFc[1+0.001xFc^2^λ^3^/sin(2θ)]^-1/4^
Primary atom site location: structure-invariant direct methods	Extinction coefficient: 0.0204 (8)
Secondary atom site location: difference Fourier map	

### Special details

**Table:**

Geometry. All e.s.d.'s (except the e.s.d. in the dihedral angle between two l.s. planes) are estimated using the full covariance matrix. The cell e.s.d.'s are taken into account individually in the estimation of e.s.d.'s in distances, angles and torsion angles; correlations between e.s.d.'s in cell parameters are only used when they are defined by crystal symmetry. An approximate (isotropic) treatment of cell e.s.d.'s is used for estimating e.s.d.'s involving l.s. planes.
Refinement. Refinement of *F*^2^ against ALL reflections. The weighted *R*-factor *wR* and goodness of fit *S* are based on *F*^2^, conventional *R*-factors *R* are based on *F*, with *F* set to zero for negative *F*^2^. The threshold expression of *F*^2^ > σ(*F*^2^) is used only for calculating *R*-factors(gt) *etc*. and is not relevant to the choice of reflections for refinement. *R*-factors based on *F*^2^ are statistically about twice as large as those based on *F*, and *R*- factors based on ALL data will be even larger.

### Fractional atomic coordinates and isotropic or equivalent isotropic displacement parameters (Å^2^)

**Table:**

	*x*	*y*	*z*	*U*_iso_*/*U*_eq_	
I	0.01644 (2)	0.271298 (12)	0.412327 (13)	0.03036 (10)	
S	0.31859 (7)	0.47908 (4)	0.80435 (4)	0.02106 (14)	
O1	0.3994 (2)	0.62669 (12)	0.55765 (13)	0.0216 (3)	
O2	0.2767 (3)	0.38039 (13)	0.79316 (14)	0.0304 (4)	
O3	0.4628 (2)	0.50442 (16)	0.86163 (15)	0.0343 (4)	
C1	0.3367 (3)	0.52624 (17)	0.68298 (18)	0.0196 (5)	
C2	0.2699 (3)	0.48622 (17)	0.59100 (18)	0.0192 (5)	
C3	0.1833 (3)	0.40340 (17)	0.56413 (18)	0.0216 (5)	
H3	0.1530	0.3570	0.6128	0.026*	
C4	0.1444 (3)	0.39292 (18)	0.46308 (19)	0.0236 (5)	
C5	0.1860 (3)	0.46045 (18)	0.39014 (19)	0.0240 (5)	
H5	0.1549	0.4497	0.3218	0.029*	
C6	0.2718 (3)	0.54330 (18)	0.41501 (18)	0.0224 (5)	
C7	0.3120 (3)	0.55133 (17)	0.51657 (18)	0.0204 (5)	
C8	0.4119 (3)	0.60993 (18)	0.65900 (18)	0.0216 (5)	
C9	0.1490 (3)	0.53904 (18)	0.85741 (18)	0.0240 (5)	
C10	0.1754 (4)	0.6275 (2)	0.9019 (2)	0.0381 (7)	
H10	0.2825	0.6548	0.9033	0.046*	
C11	0.0447 (5)	0.6749 (3)	0.9437 (3)	0.0508 (9)	
H11	0.0610	0.7355	0.9741	0.061*	
C12	−0.1097 (5)	0.6348 (3)	0.9418 (2)	0.0497 (10)	
H12	−0.1996	0.6682	0.9707	0.060*	
C13	−0.1359 (4)	0.5464 (3)	0.8984 (2)	0.0456 (9)	
H13	−0.2432	0.5193	0.8978	0.055*	
C14	−0.0047 (3)	0.4970 (2)	0.85537 (19)	0.0311 (6)	
H14	−0.0207	0.4361	0.8255	0.037*	
C15	0.3140 (4)	0.6191 (2)	0.3392 (2)	0.0308 (6)	
H15A	0.2394	0.6735	0.3474	0.046*	
H15B	0.3021	0.5933	0.2708	0.046*	
H15C	0.4279	0.6401	0.3500	0.046*	
C16	0.4982 (3)	0.6852 (2)	0.7172 (2)	0.0284 (5)	
H16A	0.6032	0.7001	0.6846	0.043*	
H16B	0.5189	0.6626	0.7861	0.043*	
H16C	0.4295	0.7426	0.7193	0.043*	

### Atomic displacement parameters (Å^2^)

**Table:**

	*U*^11^	*U*^22^	*U*^33^	*U*^12^	*U*^13^	*U*^23^
I	0.02777 (13)	0.02830 (13)	0.03502 (14)	−0.00635 (6)	0.00123 (8)	−0.00964 (7)
S	0.0220 (3)	0.0220 (3)	0.0192 (3)	0.0029 (2)	0.0004 (2)	0.0016 (2)
O1	0.0229 (8)	0.0197 (8)	0.0224 (8)	−0.0016 (7)	0.0032 (7)	0.0006 (7)
O2	0.0432 (12)	0.0204 (9)	0.0275 (9)	0.0044 (8)	0.0045 (8)	0.0035 (7)
O3	0.0264 (10)	0.0488 (12)	0.0277 (9)	−0.0001 (9)	−0.0064 (8)	0.0021 (9)
C1	0.0192 (11)	0.0205 (11)	0.0190 (11)	0.0006 (9)	0.0015 (9)	−0.0001 (9)
C2	0.0169 (10)	0.0208 (11)	0.0200 (11)	0.0020 (9)	0.0024 (8)	−0.0003 (9)
C3	0.0208 (11)	0.0198 (11)	0.0243 (11)	−0.0006 (9)	0.0030 (9)	−0.0005 (9)
C4	0.0196 (11)	0.0228 (12)	0.0285 (12)	0.0002 (9)	0.0019 (9)	−0.0051 (10)
C5	0.0243 (12)	0.0274 (12)	0.0203 (11)	0.0035 (10)	−0.0015 (9)	−0.0028 (10)
C6	0.0222 (12)	0.0232 (12)	0.0219 (11)	0.0054 (9)	0.0025 (9)	0.0018 (10)
C7	0.0178 (11)	0.0185 (11)	0.0250 (11)	0.0004 (8)	0.0027 (9)	−0.0012 (9)
C8	0.0212 (11)	0.0213 (11)	0.0222 (11)	0.0010 (9)	0.0028 (9)	−0.0005 (9)
C9	0.0296 (13)	0.0250 (12)	0.0174 (10)	0.0053 (10)	0.0046 (10)	0.0042 (9)
C10	0.0479 (18)	0.0284 (14)	0.0383 (15)	−0.0016 (13)	0.0175 (14)	−0.0031 (12)
C11	0.073 (3)	0.0323 (16)	0.0474 (19)	0.0140 (16)	0.0297 (18)	0.0001 (14)
C12	0.058 (2)	0.059 (2)	0.0323 (16)	0.0347 (18)	0.0188 (15)	0.0125 (15)
C13	0.0245 (14)	0.085 (3)	0.0269 (14)	0.0115 (15)	0.0020 (11)	0.0152 (16)
C14	0.0261 (13)	0.0458 (16)	0.0214 (12)	0.0018 (12)	−0.0038 (10)	0.0030 (11)
C15	0.0378 (15)	0.0302 (14)	0.0245 (12)	0.0014 (11)	0.0019 (11)	0.0068 (11)
C16	0.0288 (13)	0.0264 (13)	0.0299 (13)	−0.0052 (11)	0.0018 (10)	−0.0049 (11)

### Geometric parameters (Å, °)

**Table:**

I—O2^i^	3.282 (2)	C8—C16	1.481 (3)
I—C4	2.106 (3)	C9—C14	1.380 (4)
S—O2	1.433 (2)	C9—C10	1.390 (4)
S—O3	1.436 (2)	C10—C11	1.371 (4)
S—C1	1.746 (2)	C10—H10	0.9500
S—C9	1.762 (3)	C11—C12	1.374 (6)
O1—C8	1.366 (3)	C11—H11	0.9500
O1—C7	1.383 (3)	C12—C13	1.383 (6)
C1—C8	1.362 (3)	C12—H12	0.9500
C1—C2	1.444 (3)	C13—C14	1.395 (4)
C2—C7	1.388 (3)	C13—H13	0.9500
C2—C3	1.403 (3)	C14—H14	0.9500
C3—C4	1.381 (3)	C15—H15A	0.9800
C3—H3	0.9500	C15—H15B	0.9800
C4—C5	1.396 (4)	C15—H15C	0.9800
C5—C6	1.394 (4)	C16—H16A	0.9800
C5—H5	0.9500	C16—H16B	0.9800
C6—C7	1.387 (3)	C16—H16C	0.9800
C6—C15	1.504 (3)		
			
C4—I—O2^i^	165.69 (8)	O1—C8—C16	114.9 (2)
O2—S—O3	119.1 (1)	C14—C9—C10	121.8 (3)
O2—S—C1	107.0 (1)	C14—C9—S	119.7 (2)
O3—S—C1	108.7 (1)	C10—C9—S	118.5 (2)
O2—S—C9	108.5 (1)	C11—C10—C9	119.1 (3)
O3—S—C9	107.9 (1)	C11—C10—H10	120.4
C1—S—C9	104.9 (1)	C9—C10—H10	120.4
C8—O1—C7	106.9 (2)	C10—C11—C12	120.1 (3)
C8—C1—C2	107.7 (2)	C10—C11—H11	120.0
C8—C1—S	125.6 (2)	C12—C11—H11	120.0
C2—C1—S	126.6 (2)	C11—C12—C13	120.9 (3)
C7—C2—C3	119.3 (2)	C11—C12—H12	119.6
C7—C2—C1	104.5 (2)	C13—C12—H12	119.6
C3—C2—C1	136.1 (2)	C12—C13—C14	119.9 (3)
C4—C3—C2	116.5 (2)	C12—C13—H13	120.0
C4—C3—H3	121.8	C14—C13—H13	120.0
C2—C3—H3	121.8	C9—C14—C13	118.2 (3)
C3—C4—C5	122.9 (2)	C9—C14—H14	120.9
C3—C4—I	120.4 (2)	C13—C14—H14	120.9
C5—C4—I	116.7 (2)	C6—C15—H15A	109.5
C6—C5—C4	121.7 (2)	C6—C15—H15B	109.5
C6—C5—H5	119.2	H15A—C15—H15B	109.5
C4—C5—H5	119.2	C6—C15—H15C	109.5
C7—C6—C5	114.3 (2)	H15A—C15—H15C	109.5
C7—C6—C15	122.5 (2)	H15B—C15—H15C	109.5
C5—C6—C15	123.2 (2)	C8—C16—H16A	109.5
O1—C7—C2	110.6 (2)	C8—C16—H16B	109.5
O1—C7—C6	124.1 (2)	H16A—C16—H16B	109.5
C2—C7—C6	125.3 (2)	C8—C16—H16C	109.5
C1—C8—O1	110.3 (2)	H16A—C16—H16C	109.5
C1—C8—C16	134.8 (2)	H16B—C16—H16C	109.5
			
O2—S—C1—C8	−163.4 (2)	C5—C6—C7—O1	178.8 (2)
O3—S—C1—C8	−33.6 (3)	C15—C6—C7—O1	−2.7 (4)
C9—S—C1—C8	81.5 (2)	C5—C6—C7—C2	−1.7 (4)
O2—S—C1—C2	19.2 (2)	C15—C6—C7—C2	176.8 (2)
O3—S—C1—C2	149.0 (2)	C2—C1—C8—O1	−0.4 (3)
C9—S—C1—C2	−95.8 (2)	S—C1—C8—O1	−178.2 (2)
C8—C1—C2—C7	0.1 (3)	C2—C1—C8—C16	178.1 (3)
S—C1—C2—C7	177.8 (2)	S—C1—C8—C16	0.3 (4)
C8—C1—C2—C3	178.8 (3)	C7—O1—C8—C1	0.5 (3)
S—C1—C2—C3	−3.4 (4)	C7—O1—C8—C16	−178.3 (2)
C7—C2—C3—C4	−0.4 (3)	O2—S—C9—C14	−17.4 (2)
C1—C2—C3—C4	−179.1 (3)	O3—S—C9—C14	−147.6 (2)
C2—C3—C4—C5	−0.7 (4)	C1—S—C9—C14	96.7 (2)
C2—C3—C4—I	179.5 (2)	O2—S—C9—C10	161.6 (2)
C3—C4—C5—C6	0.7 (4)	O3—S—C9—C10	31.4 (2)
I—C4—C5—C6	−179.5 (2)	C1—S—C9—C10	−84.4 (2)
C4—C5—C6—C7	0.5 (4)	C14—C9—C10—C11	−0.9 (5)
C4—C5—C6—C15	−178.0 (2)	S—C9—C10—C11	−179.8 (3)
C8—O1—C7—C2	−0.5 (3)	C9—C10—C11—C12	0.3 (5)
C8—O1—C7—C6	179.0 (2)	C10—C11—C12—C13	0.3 (5)
C3—C2—C7—O1	−178.7 (2)	C11—C12—C13—C14	−0.3 (5)
C1—C2—C7—O1	0.3 (3)	C10—C9—C14—C13	0.8 (4)
C3—C2—C7—C6	1.7 (4)	S—C9—C14—C13	179.8 (2)
C1—C2—C7—C6	−179.3 (2)	C12—C13—C14—C9	−0.3 (4)

Symmetry codes: (i) *x*−1/2, −*y*+1/2, *z*−1/2.

### Hydrogen-bond geometry (Å, °)

**Table:**

*D*—H···*A*	*D*—H	H···*A*	*D*···*A*	*D*—H···*A*
C11—H11···O1^ii^	0.95	2.59	3.382 (4)	141
C13—H13···O3^iii^	0.95	2.44	3.342 (4)	159
C14—H14···O2	0.95	2.58	2.931 (4)	103
C16—H16B···O3	0.98	2.48	3.191 (4)	129

Symmetry codes: (ii) *x*−1/2, −*y*+3/2, *z*+1/2; (iii) *x*−1, *y*, *z*.
